# Addressing the poverty–nutrition gap for SDG 1 and SDG 2: a Bayesian geo-additive analysis of spatial heterogeneity and nonlinear maternal risks in childhood stunting in India (NFHS-4 and NFHS-5)

**DOI:** 10.3389/fpubh.2026.1854301

**Published:** 2026-08-03

**Authors:** V. Vinay Shankar, G. Rithanya Grishma, R. Niveditha, K. Sathya Narayana Sharma, Saralees Nadarajah, S. Yashuvanthra Dhevi, J. E. Yuvaraj Kumar, G. S. Heerthihaa

**Affiliations:** 1 Department of Mathematics, School of Advanced Sciences, Vellore Institute of Technology, Vellore, India; 2 Department of Mathematics, University of Manchester, Manchester, United Kingdom

**Keywords:** Bayesian modeling, childhood stunting, district-level analysis, geo-additive models, maternal nutrition, NFHS, spatial epidemiology

## Abstract

**Introduction:**

Childhood stunting remains a critical indicator of chronic undernutrition and a major barrier to achieving Sustainable Development Goals 1 and 2. Despite gradual improvements, substantial geographic disparities in stunting risk persist across India. This study characterised the spatial and temporal heterogeneity of childhood stunting using harmonized district-level data from NFHS-4 (2015-16) and NFHS-5 (2019-21).

**Methods:**

A Bayesian spatial geo-additive logistic regression model was applied to 482,960 children aged 0-59 months from 575 harmonized districts. The framework incorporated district-level BYM2 spatial random effects, district-specific survey-round interaction effects, and RW2 smoothers for child age and maternal BMI, estimated using Integrated Nested Laplace Approximation (INLA).

**Results:**

The spatial interaction model demonstrated substantially improved fit over the non-spatial model (ΔWAIC = 4,105.2), with a high estimated spatial fraction (φ = 0.983; 95% CrI: 0.938-0.999), indicating that most residual district-level variation was spatially structured. Model predictions showed strong agreement with weighted survey prevalence estimates (Pearson *r* = 0.975; *R*^2^ = 0.950). After accounting for district-specific temporal interactions, there was no evidence of a uniform national change in stunting risk between survey rounds (AOR = 0.986; 95% CrI: 0.960–1.013), with substantial heterogeneity in district-level temporal trajectories. Stunting risk peaked between 18 and 24 months of age and was elevated among children of severely undernourished mothers. Based on 95% posterior credible intervals of district-specific survey-round interaction effects, 59 districts demonstrated credible improvement, 35 demonstrated credible worsening, and 481 showed no credible evidence of change. A joint dOR-prevalence framework identified 213 districts (37.0%) as highest-priority areas, combining elevated adjusted district-level risk with high absolute stunting burden.

**Discussion:**

These findings demonstrate that childhood stunting in India remains strongly patterned by geography, with temporal changes varying substantially across districts. Geographically targeted and maternal-focused interventions will be essential for achieving equitable reductions in stunting and accelerating progress toward national nutrition goals.

## 1 Introduction

Childhood stunting, defined as low height-for-age, remains a key indicator of chronic undernutrition and a major barrier to achieving Sustainable Development Goal (SDG) 2: Zero Hunger by 2030. The resulting physiological imbalance reflects prolonged exposure to adverse socioeconomic conditions, highlighting the strong linkage between undernutrition and the structural poverty addressed under SDG 1: No Poverty. The interaction between these goals forms a reinforcing cycle in which household income constraints limit access to adequate nutrition, healthcare, and hygienic living environments, ultimately resulting in persistent nutritional deficiencies that impair child growth. Consequently, the effects of stunting extend far beyond childhood, influencing survival, cognitive development, educational attainment, and long-term economic productivity (1–4). Despite substantial global and national interventions, childhood stunting continues to affect millions of children in low- and middle-income countries, including India, where it remains a significant public health and developmental concern (5–9).

Data from recent rounds of the National Family Health Survey (NFHS)—NFHS-4 (2015–16) and NFHS-5 (2019–21)—indicate a gradual decline in the overall national prevalence of childhood stunting. However, these aggregate improvements often conceal persistent geographic and socioeconomic disparities (10–15). Previous research has identified considerable heterogeneity in child nutritional outcomes across states, districts, and smaller administrative units, shaped by interactions among environmental exposures, socio-demographic conditions, and access to essential health and nutrition services (16–22). Such spatial inequalities highlight the importance of district-level investigations that can reveal localized patterns of progress or stagnation.

A large body of literature has documented the association between childhood stunting and multiple household- and community-level determinants, including economic status, maternal education, sanitation, dietary diversity, environmental conditions, and maternal health (23–31). Biological and environmental determinants also influence growth trajectories across childhood and adolescence (32). During the school-age period (5–19 years), favorable conditions can partially offset early-life nutritional deficits and reinforce early developmental progress (33). Maternal characteristics, particularly adolescent pregnancy, represent another important pathway influencing childhood undernutrition. Adolescent mothers are more likely to experience undernutrition, anemia, lower educational attainment, and limited access to healthcare, which together increase the risk of stunting among their children (34). National estimates further demonstrate the magnitude of the problem in India: in 2017, malnutrition contributed to 68.2% of deaths among children under five, with maternal anemia affecting 54.4% of women and child stunting affecting 39.3% of children. These burdens are particularly pronounced in states such as Uttar Pradesh and Bihar, and projections suggest that national targets for reducing stunting may remain unmet without stronger multisectoral interventions (35).

Although many studies have examined determinants of childhood stunting, much of the existing analytical work relies on conventional regression approaches that assume linear relationships and spatial independence. These assumptions are increasingly recognized as unrealistic in the context of undernutrition research (36, 37). Evidence indicates that the risk of stunting varies nonlinearly with child age—particularly during the critical first two years of life—and that geographically proximate districts often share contextual influences that induce spatial autocorrelation in outcomes (38–40). Ignoring these nonlinear and spatial dependencies can lead to biased estimates and reduced explanatory power.

To address such limitations, recent methodological advances have incorporated spatial, multilevel, and Bayesian modeling approaches. In particular, Bayesian geo-additive models allow the simultaneous estimation of nonlinear covariate effects, spatial dependence structures, and unobserved district-level heterogeneity (41–44). Applications of these frameworks in India and other settings have revealed meaningful geographic clustering of childhood undernutrition and have demonstrated improved model performance relative to non-spatial alternatives (45, 46).

Despite these advances, several important gaps remain in the literature. Existing studies often focus on a single NFHS round, limiting their ability to assess temporal changes in spatial risk patterns. Other analyses operate at national or state levels, masking substantial variation within smaller administrative units. Furthermore, critical biological predictors such as child age and maternal body mass index (BMI) are frequently modeled as linear covariates, even though empirical evidence suggests that their associations with stunting are inherently nonlinear. Few studies therefore provide an integrated modeling framework capable of simultaneously capturing nonlinear biological effects, district-level spatial heterogeneity, and district-specific temporal variation across survey rounds.

This study is primarily epidemiological in focus, aimed at characterizing the geographic distribution and temporal dynamics of childhood stunting across Indian districts. A Bayesian spatial geo-additive modeling framework serves as the analytical vehicle to enable rigorous estimation of these patterns. The primary objectives were: (i) to quantify district-level spatial heterogeneity in childhood stunting across India; and (ii) to evaluate district-specific temporal changes in stunting risk between NFHS-4 (2015–16) and NFHS-5 (2019–21) using a unified Bayesian spatial interaction model. Secondary objectives were: (iii) to characterize the nonlinear association between child age and childhood stunting; and (iv) to evaluate the nonlinear association between maternal BMI and stunting risk. In addition, district-level spatial odds ratios (dORs) and a complementary dOR–prevalence framework were used to identify districts with elevated adjusted risk and high absolute burden for policy prioritization.

To address these objectives, harmonized district-level data from NFHS-4 (2015–16) and NFHS-5 (2019–21) were analyzed using a Bayesian spatial geo-additive framework incorporating BYM2 spatial random effects, RW2 nonlinear smoothers, and district-specific survey-round interaction effects (see Section 4 for full model specification). Together, these analyses provide policy-relevant evidence to support geographically targeted resource allocation and nutrition programme prioritization aimed at reducing childhood stunting in India. A summary of existing studies, their methodologies, and key limitations is presented in Table 1.

**Table 1:** Summary of the existing work.

Author(s)	Source	Methodology	Key limitations
Srivastava et al. (7)	NFHS 1998–2016	Small Area Estimation (SAE); district-level mapping	No NFHS-5; no nonlinear effects; non-Bayesian; no spatial random effects (BYM2).
BS and Guddattu (11)	NFHS-4 & NFHS-5	District-level logistic regression to compare rounds	No spatial modeling; ignored spatial dependence; no nonlinear modeling.
Vennam et al. (14)	NFHS district data (640 districts)	Moran's I, LISA – exploratory spatial autocorrelation	Not a full spatial model; no Bayesian estimation; no nonlinear effects; no temporal comparison.
Das (25)	NFHS-4 & NFHS-5 district-level	Spatial regression models	Spatial yes, but no geo-additive nonlinear effects; no pooled NFHS-4/NFHS-5 modeling.
Bhadra (43)	NFHS-5	Copula-based geo-additive Bayesian model	Cross-sectional only (NFHS-5); no NFHS-4 comparison; no district transition analysis.
Padigapati et al. (40)	NFHS-5	Spatial + multilevel modeling	No temporal comparison; nonlinear effects not modeled; no BYM2 spatial structure.

## 2 Methods

The analytical workflow integrated harmonized NFHS-4 and NFHS-5 data, district harmonization procedures, Bayesian model estimation, and policy-oriented district prioritization metrics. Detailed model specifications are provided in Section 4.

### 2.1 Data source

This study utilized data from the National Family Health Survey (NFHS), a nationally representative Demographic and Health Survey conducted under the stewardship of the Ministry of Health and Family Welfare, Government of India. Specifically, data from NFHS-4 (2015–16) and NFHS-5 (2019–21) were used for the present analysis.

Although earlier NFHS rounds provide long-term national trends, district-level spatially disaggregated data with consistent boundaries are available only from NFHS-4 onwards. Accordingly, the analysis was restricted to NFHS-4 and NFHS-5 to ensure spatial and temporal comparability, particularly for district-level spatial modeling.

The surveys were implemented by the International Institute for Population Sciences (IIPS), which obtained ethical approval for data collection and dissemination. The datasets used are publicly available and fully anonymized; therefore, no additional ethical approval was required for this study.

### 2.2 Sampling design

Both NFHS-4 and NFHS-5 employed a stratified two-stage sampling design in rural and urban areas. In rural areas, villages were selected as primary sampling units (PSUs) using probability proportional to size (PPS) sampling. In urban areas, census enumeration blocks served as PSUs.

Households within each PSU were selected using systematic random sampling. NFHS-5 was designed to produce reliable estimates at the district, state/union territory, and national levels, covering 707 districts as of March 2017. Detailed sampling procedures are documented in the official NFHS reports.

### 2.3 Study population

The study population comprised children aged 0–59 months with valid anthropometric measurements. The pooled dataset initially contained 492,547 children (NFHS-4: 259,627; NFHS-5: 232,920). After excluding 9,587 observations (1.95%) with missing covariate information — attributable entirely to unavailable maternal BMI measurements, as all other analytical variables were complete — 482,960 complete cases were retained for analysis. Missingness was not spatially concentrated: only 18 of the 772 districts represented in the pooled raw dataset exhibited exclusion rates exceeding 10% and district-level exclusion rates ranged from 0.0 to 44.5%, with the upper extreme reflecting small, geographically remote districts.

To ensure valid spatial and temporal comparisons, the analysis was restricted to districts common to both NFHS-4 and NFHS-5. Following district harmonization and spatial alignment procedures, 575 districts remained for analysis and formed the basis of all subsequent spatial and temporal modeling.

## 3 Data preparation and district harmonization

### 3.1 District harmonization and spatial unit alignment

The analysis was restricted to districts common to both NFHS-4 (2015–16) and NFHS-5 (2019–21) to ensure temporal and spatial comparability. Due to the absence of a nationally harmonized district identifier linking NFHS survey records directly to Census 2011 administrative boundaries, spatial units were aligned using the DHSREGCO district identifiers available within the NFHS datasets. District polygons from the Census 2011 shapefile were linked to survey districts through a spatial intersection of NFHS-4 cluster point locations with census polygon boundaries, with the modal DHSREGCO code assigned to each polygon. All 641 Census 2011 district polygons were successfully matched through this spatial linkage procedure, and no nearest-neighbor fallback assignments were required. One district code (DHSREGCO = 10) was absent following polygon deduplication. To preserve one-to-one correspondence between survey districts and spatial units, the code was assigned to the largest remaining unmatched polygon. Following harmonization and restriction to districts common to both survey rounds, 575 districts remained for analysis.

Because all Census 2011 polygons were successfully matched through spatial intersection and no alternative district assignments were required, additional harmonization sensitivity analyses were not considered necessary. The harmonization procedure therefore involved minimal ambiguity in the allocation of survey districts to spatial units.

District-level spatial adjacency was constructed using Census 2011 district boundary shapefiles and a queen contiguity criterion with a 100 m snapping tolerance. The resulting adjacency graph contained 10 disconnected sub-graphs, corresponding to eight geographically isolated districts (Dhalai, South Garo Hills, Porbandar, Amreli, Anand, Diu, Lakshadweep, and Nicobars). Districts forming isolated spatial units or disconnected subgraphs were retained in the analysis. The Besag–York–Mollié 2 (BYM2) specification accommodates such structures through the inclusion of an unstructured random-effect component, ensuring model stability in the presence of sparse or disconnected neighborhood structures. For these isolated districts, spatial effects are estimated primarily through the unstructured BYM2 component rather than the intrinsic conditional autoregressive (ICAR) component.

### 3.2 Outcome variable

The primary outcome was childhood stunting, defined as height-for-age z-score (HAZ) < −2 standard deviations based on the WHO Child Growth Standards. Stunting reflects chronic undernutrition and cumulative exposure to adverse socioeconomic and environmental conditions during early childhood.

The outcome variable was binary and defined as:


$$Y_{ij}=\{\begin{array}{cc} \begin{array}{c} 1,\\ 0, \end{array} & \begin{array}{c} f\;child\;i\;in\;district\;j\;is\;stunted\\ otherwise \end{array} \end{array}$$

### 3.3 Explanatory variables

Based on prior empirical evidence and data availability, the following covariates were included in the analysis.

#### 3.3.1 Child-level variables

Child age (months) was included as a continuous measure of age during early childhood. Child age was derived following standard Demographic and Health Survey guidelines. For NFHS-4, age was calculated as the difference between the interview date and date of birth, whereas the survey-provided age variable was used directly in NFHS-5. Both approaches yield equivalent measures and are routinely employed in DHS-based analyses. Child sex was included as a fixed-effect covariate.

#### 3.3.2 Maternal variables

Maternal educational attainment was coded as a categorical (factor) variable with four levels (no education, primary, secondary, higher education), with no education as the reference category. Maternal body mass index (BMI, kg/m^2^) was included as an indicator of maternal nutritional status.

#### 3.3.3 Socioeconomic and survey variables

Household wealth quintile was obtained from the NFHS wealth index (v190), which is constructed using principal components analysis (PCA) of household assets, housing characteristics, and access to basic amenities. The resulting index is categorized into five quintiles ranging from poorest to richest and was entered into the model as a categorical (factor) covariate, with the poorest quintile as the reference category, consistent with the treatment of maternal education described above. This is a household-level variable; consequently, households within the same district may belong to different wealth quintile categories and the measure should not be interpreted as a district-level aggregate. Survey round was represented by a binary indicator variable distinguishing NFHS-4 (2015–16; coded 0) and NFHS-5 (2019–21; coded 1). This variable was included as a fixed effect to estimate the overall temporal contrast in childhood stunting between the two survey rounds, while permitting district-specific deviations from this overall contrast through an interaction with district (see Section 4.1).

## 4 Statistical analysis

### 4.1 Bayesian spatial geo-additive model

A Bayesian spatial geo-additive logistic regression model was employed to examine the determinants and spatial heterogeneity of childhood stunting.

### 4.2 Model specification

Let *Y*_*ij*_ denote the stunting status of child *i* residing in district *j*. The response variable was defined as a binary outcome, where *Y*_*ij*_ = 1 if the child was stunted and *Y*_*ij*_ = 0 otherwise. The outcome was assumed to follow a Bernoulli distribution:


$$\begin{array}{l} Y_{ij}\sim Bernoulli\left(p_{ij}\right) \end{array}$$

where *p*_*ij*_ represents the probability that child *i* in district *j* is stunted. The relationship between the covariates and the probability of stunting was modeled using a logit link function:


$$\begin{array}{l} logit\left(p_{ij}\right)=\eta_{ij} \end{array}$$

The linear predictor η_*ij*_ was specified as:


$$\begin{array}{l} \eta_{ij}=\beta_{0}+f_{1}(Age_{ij})+f_{2}(BMI_{ij})+\beta^{⊤}X_{ij}+\gamma Survey_{ij}\\ \;+\;b_{j}+\;\delta_{j}Survey\;ij \end{array}$$

where:

- β_0_ is the intercept
- *f*_1_(·) and *f*_2_(·) are nonlinear smooth functions of child age and maternal body mass index, respectively, modeled using second-order random walk (RW2) priors, a flexible semiparametric approach for capturing nonlinear covariate effects (47).
- *X*_*ij*_ is a vector of fixed-effect covariates, including household wealth quintile (categorical), maternal education (categorical), and child sex.
- β is the corresponding vector of regression coefficients.
- *Survey*_*ij*_ is a binary indicator variable identifying the survey round for child i in district j (0 = NFHS-4, 2015–16; 1 = NFHS-5, 2019–21). The survey-round variable is a standard fixed-effect covariate and could equivalently be incorporated within the fixed-effects vector X; it is presented separately here solely for notational clarity because it also appears in the district-specific interaction term below. The corresponding coefficient γ estimates the overall temporal difference in childhood stunting between the two survey rounds, after adjustment for the remaining model components.
- *b*_*j*_ represents the combined district-level Besag–York–Mollié 2 (BYM2) spatial random effect, decomposed into its structured and unstructured components in Section 4.2.
- δ_*j*_ is the district-specific survey-round interaction effect, representing the district-specific deviation from the overall survey-round effect and allowing the temporal change in childhood stunting between NFHS-4 (2015–16) and NFHS-5 (2019–21) to vary across districts. δ_*j*_ was modeled as an unstructured, independent and identically distributed (iid) Gaussian random effect indexed jointly by district and survey round (Type I space–time interaction) (48).

### 4.3 Spatial random effects specification

District-level spatial effects were modeled using the Besag–York–Mollié 2 (BYM2) formulation, which decomposes spatial variability into structured and unstructured components while providing an interpretable parameter governing the proportion of variance attributable to spatial dependence (49):


$$\begin{array}{l} b_{j}=\frac{1}{\sqrt{\tau }}\left(\sqrt{\phi }u_{j}+\sqrt{1-\phi }v_{j}\right) \end{array}$$

where:

- *b*_*j*_ denotes the combined (total) district-level spatial random effect entering the linear predictor in Section 4.1.
- *u*_*j*_ denotes the spatially structured component, modeled using an intrinsic conditional autoregressive (ICAR) prior based on district adjacency.
- *v*_*j*_ denotes the unstructured component, modeled as independent and identically distributed (iid) Gaussian noise.
- ϕ∈[0, 1] is the spatial fraction parameter representing the proportion of total variance attributable to spatial structure.
- τ denotes the overall precision parameter of the BYM2 random effect, with larger values corresponding to lower overall variance.

Following the approach of Flaibani et al. (50), the BYM2 random effect was algebraically decomposed into structured and unstructured components to enable separate evaluation of spatially correlated and locally idiosyncratic district-level variation. In the standard R-INLA implementation of the BYM2 model, the combined district-level effect (*b*_*j*_) and the scaled structured (ICAR) component (*u*_*j*_) are returned directly, whereas the unstructured component (*v*_*j*_) is not. Accordingly, the unstructured component was obtained algebraically from the above decomposition using the posterior means of *b*_*j*_, *u*_*j*_, ϕ, and τ. The resulting structured and unstructured components were visualized separately in Supplementary Figures S10, S11.

Default diffuse priors were assigned to the fixed-effect regression coefficients. For the BYM2 precision and mixing parameters, the model's default penalized complexity (PC) hyperpriors were retained, comprising a PC prior on the precision parameter (*P*(σ>1) = 0.01) and a PC prior on the spatial mixing fraction ϕ, consistent with standard Bayesian disease-mapping practice.

Household wealth quintile and maternal education capture complementary dimensions of socioeconomic status and were therefore retained simultaneously in the model. Multicollinearity among the fixed-effect covariates was assessed using variance inflation factors (VIFs) computed from an auxiliary regression, with wealth quintile and maternal education entered in their original continuous coding for diagnostic purposes because VIF is conventionally interpreted per coefficient rather than per categorical level. This auxiliary analysis did not affect the categorical specification used in the primary model. All VIF values were well below the conventional threshold of 5 (household wealth quintile: 1.331; maternal education: 1.351; child sex: 1.000; survey round: 1.022), indicating negligible multicollinearity (Supplementary Table S1).

Concurvity between the nonlinear smooth terms for child age and maternal BMI was assessed using generalized additive model diagnostics. Worst-case concurvity values were 0.012 for child age and 0.125 for maternal BMI, indicating negligible redundancy between the smooth terms. Although the combined parametric (fixed-effect) block showed a higher worst-case concurvity estimate (0.863), pairwise concurvity between the fixed-effect block and each individual smooth term was negligible (0.000), indicating no meaningful confounding between the linear and nonlinear components of the model.

### 4.4 Estimation and model assessment

All models were estimated using the Integrated Nested Laplace Approximation (INLA) framework, an efficient Bayesian inference approach for latent Gaussian models that provides accurate posterior approximations with substantially lower computational burden than traditional Markov chain Monte Carlo methods (51). Competing model specifications included a non-spatial model, a spatial BYM2 model, and a spatial interaction model incorporating district-specific survey-round effects. Model performance was evaluated using the Watanabe–Akaike information criterion (WAIC) and the Deviance Information Criterion (DIC), with lower values indicating improved model fit.

Exponentiated posterior means of fixed effects were reported as adjusted odds ratios (AORs) together with 95% Bayesian credible intervals. The spatial fraction parameter (ϕ) from the BYM2 model was used to quantify the relative contribution of structured and unstructured spatial variation to total district-level variance. Values of ϕ approaching 1 indicate that residual district-level variation is predominantly spatially structured, whereas values approaching 0 indicate predominantly unstructured heterogeneity.

As a supplementary calibration assessment, district-level model-predicted stunting probabilities were compared against direct survey-weighted prevalence estimates across all 575 harmonized districts. Agreement between model predictions and observed prevalence was evaluated using Pearson's correlation coefficient and the coefficient of determination (*R*^2^), and the corresponding scatter plot is presented in Supplementary Figure S1.

For the national comparison figure, predicted probabilities were averaged across observations within each survey round. Uncertainty was summarized using the mean lower and upper bounds of individual-level 95% posterior credible intervals, reflecting average prediction uncertainty rather than uncertainty around the population mean.

NFHS sampling weights (variable v005), provided as integers scaled by 10^6^, were rescaled and normalized within each survey round to unit mean prior to model fitting. The normalized weights were incorporated into the INLA likelihood as observation-level weights across all model specifications to improve population representativeness. However, this approach does not constitute full design-based inference. Although NFHS employs a stratified multistage cluster sampling design, variance estimation accounting for stratification and primary sampling unit clustering was not implemented within the INLA framework; the implications of this approximation are discussed in the Limitations section.

Maternal BMI was missing for 1.31% of NFHS-4 observations and 2.66% of NFHS-5 observations. Missingness patterns were assessed using logistic regression and district-level exclusion summaries. Although the missing-at-random (MAR) assumption cannot be formally verified from observed data, the low overall proportion of missing values and the absence of strong geographic concentration of exclusions were considered broadly consistent with MAR. BMI values outside the physiologically plausible range ( ≤ 0 or ≥60 kg/m^2^), corresponding to DHS non-response codes, were treated as missing and excluded prior to analysis.

### 4.5 Spatial risk mapping and transition analysis

District-level spatial odds ratios (dORs) were derived as:

*dOR*_*j*_ = exp(*b*_*j*_) where *b*_*j*_ denotes the posterior mean combined district-level spatial effect from the BYM2 model, and *dOR*_*j*_ represents the multiplicative change in the adjusted odds of childhood stunting for district *j* relative to the national baseline after adjustment for observed covariates.

Temporal change was evaluated within a unified modeling framework through district-specific survey-round interaction effects, allowing district-level changes between NFHS-4 and NFHS-5 to be estimated directly rather than inferred from separate survey-round models. For each district, posterior means and 95% Bayesian credible intervals of the interaction effect were extracted to quantify temporal change in adjusted stunting risk.

Districts were classified according to the posterior uncertainty associated with the district-specific interaction effects:

- Improved: upper bound of the 95% credible interval below 0
- Worsened: lower bound of the 95% credible interval above 0
- No credible change: 95% credible interval includes 0

This classification framework explicitly incorporates estimation uncertainty and provides probabilistic evidence regarding district-level temporal change. Districts classified as improved or worsened therefore demonstrated credible evidence of change between NFHS-4 and NFHS-5 after adjustment for observed covariates and spatial effects, whereas districts classified as showing no credible change lacked sufficient evidence to support either improvement or deterioration.

To provide a complementary policy-oriented perspective, district-level spatial odds ratios were combined with district-level stunting prevalence estimates from NFHS-5. Districts were assigned to one of four dOR–prevalence quadrants based on whether their adjusted district odds ratio exceeded 1, indicating above-average adjusted risk, and whether their observed stunting prevalence exceeded the national median across districts:

- High dOR & High Prevalence (highest-priority districts)
- High dOR & Low Prevalence
- Low dOR & High Prevalence
- Low dOR & Low Prevalence

This integrated framework captures both adjusted relative vulnerability and absolute stunting burden, providing a more nuanced basis for identifying districts requiring intensified intervention.

### 4.6 Software

All analyses were conducted using R (version ≥ 4.2). Bayesian models were fitted using the INLA package; spatial data were processed using sf and spdep, and maps were generated using tmap.

## 5 Results

### 5.1 Sample characteristics and analytic population

After harmonizing NFHS-4 (2015–16) and NFHS-5 (2019–21) and restricting the analysis to districts common to both survey rounds, a pooled dataset of children aged 0–59 months was obtained. Of the 492,547 pooled records, 9,587 (1.95%) were excluded due to missing maternal BMI measurements, the only analytical variable with incomplete data, yielding a final analytic sample of 482,960 complete cases. Restriction to districts common to both survey rounds ensured spatial and temporal comparability and resulted in 575 harmonized districts for analysis. The final analytic sample comprised 482,960 children (NFHS-4: 256,226; NFHS-5: 226,734).

Descriptive epidemiologic characteristics of the pooled sample prior to complete-case exclusion are presented in Table 2. Supplementary Figure S2 presents the model-based national comparison of childhood stunting risk between NFHS-4 and NFHS-5 derived from the district-specific survey-round interaction model.

**Table 2:** Descriptive epidemiologic characteristics of the pooled NFHS sample across NFHS-4 and NFHS-5.

Characteristic	NFHS-4 (*n* = 259,627)	NFHS-5 (*n* = 232,920)	Total (*n* = 492,547)
Stunted, weighted % (survey-weighted prevalence)	33.1%	31.0%	32.1%
Male sex, *n* (%)	52.0%	51.8%	51.9%
Child age, months: mean (SD)	29.8 (17.2)	29.7 (17.5)	29.7 (17.3)
Maternal BMI, kg/m^2^: mean (SD)	21.20 (3.75)	21.85 (3.98)	21.51 (3.87)
Maternal BMI missing (%)	1.31	2.66	1.95
Household wealth quintile	Mean 2.9 (1.4)	Mean 3.1 (1.4)	Mean 3.0 (1.4)
Maternal education level	Mean 1.8 (1.1)	Mean 2.0 (1.1)	Mean 1.9 (1.1)

Descriptive statistics are based on the pooled sample before exclusion of observations with missing maternal BMI. The final analytic sample used for modeling comprised 482,960 children.

### 5.2 Model comparison and spatial structure

Table 3 compares the non-spatial logistic regression model, the spatial BYM2 model, and the Bayesian spatial interaction model incorporating district-specific survey-round interaction effects. The spatial interaction model demonstrated the best overall fit, with a reduction in WAIC of 4,105.2 points compared with the non-spatial model (WAIC: 501,505.2 vs. 505,610.4). Relative to the spatial BYM2 model without interactions (WAIC: 502,357.7), inclusion of district-specific survey-round interaction effects further improved model fit by 852.4 WAIC points, indicating substantial heterogeneity in temporal change across districts.

**Table 3:** Comparison of non-spatial, spatial BYM2, and district-specific survey-round interaction models using WAIC and DIC.

Model	WAIC	DIC	ΔWAIC	ΔDIC
Non-spatial	505610.4	505511.8	4105.2	4681.71
Spatial BYM2, no interaction	502357.7	501965.3	852.45	1135.20
Spatial BYM2 + district × survey-round interaction	501505.2	500830.1	0.00	0.00

The spatial heterogeneity parameters from the final Bayesian spatial interaction model are reported in Table 4. The estimated spatial fraction was extremely high (ϕ = 0.983; 95% CrI: 0.938–0.999), indicating that residual district-level variation was predominantly attributable to the structured spatial component rather than unstructured heterogeneity. This finding confirms strong spatial autocorrelation in childhood stunting risk across districts, with geographically proximate districts tending to share similar adjusted risk profiles beyond what is explained by the observed covariates.

**Table 4:** Posterior hyperparameter estimates from the Bayesian spatial interaction model.

Hyperparameter	Posterior mean	95% CrI LCI	95% CrI UCI
Precision for child age (b19)	1.468	0.719	2.716
Precision for maternal BMI	20.926	3.792	63.846
Precision for district effect (τ)	20.402	15.147	27.025
Spatial fraction (ϕ)	0.983	0.938	0.999
Precision for district × survey-round interaction	32.890	28.000	38.384

District-level model predictions showed excellent agreement with weighted survey prevalence estimates (Pearson *r* = 0.975; *R*^2^ = 0.950; Supplementary Figure S1), demonstrating good calibration of the final model. Model comparison based on the Deviance Information Criterion (DIC) yielded similar conclusions, with the spatial interaction model outperforming both the non-spatial and spatial-only alternatives.

Prior sensitivity was assessed by refitting the spatial model with two alternative penalized complexity (PC) hyperpriors that diverged from the default in opposite directions—one favoring lower values of ϕ (greater unstructured variation) and one favoring higher values of ϕ (greater spatially structured variation). Both alternatives produced negligible changes relative to the default specification (Δϕ ≤ 0.004; ΔWAIC ≤ 0.15), indicating that the principal findings were robust to prior specification across a genuinely contrasting range of plausible priors.

It should be noted that the spatial adjacency matrix contained 10 disconnected sub-graphs, primarily corresponding to geographically isolated districts and island territories. Although the BYM2 formulation accommodates such structures through its unstructured component, the estimated spatial fraction should be interpreted with this limitation in mind.

Collectively, these findings provide strong support for the inclusion of district-level spatial structure and district-specific temporal effects when modeling childhood stunting across India.

### 5.3 Fixed effects: socioeconomic and temporal determinants of stunting

Adjusted odds ratios (AORs) with 95% Bayesian credible intervals from the Bayesian spatial interaction model are presented in Table 5.

**Table 5:** Fixed-effect adjusted odds ratios (AORs) and 95% Bayesian credible intervals from the spatial interaction model.

Variable	AOR	95% LCI	95% UCI
Intercept	0.605	0.561	0.653
Household wealth quintile			
Poorer vs. poorest	0.885	0.868	0.903
Middle vs. poorest	0.769	0.752	0.787
Richer vs. poorest	0.633	0.617	0.649
Richest vs. poorest	0.518	0.502	0.535
Maternal education			
Primary vs. no education	0.921	0.901	0.942
Secondary vs. no education	0.770	0.756	0.784
Higher vs. no education	0.593	0.575	0.611
Sex (male)9	1.048	1.034	1.062
Survey round (NFHS-5 = 1)	0.986	0.960	1.013

Household wealth quintile and maternal education were modeled as categorical fixed-effect covariates. Odds ratios for wealth are interpreted relative to the poorest wealth quintile, while odds ratios for maternal education are interpreted relative to no formal education. Reference categories were female child for sex and NFHS-4 (2015–16) for survey round.

Higher household wealth was strongly associated with lower odds of childhood stunting, with the magnitude of the association increasing across successive quintiles. Relative to the poorest quintile, the adjusted odds of stunting were 11.5% lower in the poorer quintile (AOR = 0.885; 95% CrI: 0.868–0.903), 23.1% lower in the middle quintile (AOR = 0.769; 95% CrI: 0.752–0.787), 36.7% lower in the richer quintile (AOR = 0.633; 95% CrI: 0.617–0.649), and 48.2% lower in the richest quintile (AOR = 0.518; 95% CrI: 0.502–0.535). A similar pattern was observed for maternal education: relative to children of mothers with no education, the adjusted odds of stunting were 7.9% lower among children of mothers with primary education (AOR = 0.921; 95% CrI: 0.901–0.942), 23.0% lower with secondary education (AOR = 0.770; 95% CrI: 0.756–0.784), and 40.7% lower with higher education (AOR = 0.593; 95% CrI: 0.575–0.611). Male children exhibited modestly higher odds of stunting than female children (AOR = 1.048; 95% CrI: 1.034–1.062).

After accounting for district-specific survey-round interaction effects, the overall survey-round effect was close to the null (AOR = 0.986; 95% CrI: 0.960–1.013), indicating little evidence of a uniform national change in childhood stunting between NFHS-4 and NFHS-5. Instead, temporal changes varied substantially across districts.

Overall, these results indicate that socioeconomic inequalities remain major determinants of childhood stunting, with effects that strengthen disproportionately at higher levels of wealth and maternal education, while temporal patterns vary substantially across geographic areas.

### 5.4 Nonlinear effects of child age and maternal BMI

Supplementary Figures S3, S4 present the estimated nonlinear effects of child age and maternal body mass index (BMI) on the predicted probability of childhood stunting, derived from second-order random walk (RW2) smoothers and expressed on the probability scale at the population-weighted average covariate profile, with 95% posterior credible intervals across the full observed range of each covariate. Pronounced nonlinear associations were observed for both covariates.

To evaluate the contribution of each nonlinear term, reduced versions of the Bayesian spatial interaction model were fitted in which the RW2 smoother for a given covariate was replaced with a linear effect while retaining the remaining model components, including the BYM2 spatial effect and district-specific survey-round interaction terms. Relative to the full model, replacing the age smoother with a linear term increased WAIC by 7,086.7, whereas replacing the BMI smoother with a linear term increased WAIC by 191.2. These results indicate that both nonlinear terms contribute substantially to model fit beyond a linear specification, with the nonlinear effect of child age providing a considerably larger improvement than that of maternal BMI.

### 5.5 Child age

The predicted probability of stunting by child age is shown in Supplementary Figure S3. Stunting risk increased rapidly during the first two years of life, reaching a peak between approximately 18 and 24 months of age. At the peak (around 22 months), the predicted probability of stunting was 37.5% (95% CrI: 35.6%−39.4%) at the population-weighted average covariate profile, compared with 32.5% at the population-average age effect. Stunting risk declined after the peak, with some stabilization in mid-childhood, and a smaller secondary elevation was observed around 38–42 months of age; however, the associated credible intervals were wider at older ages, indicating greater uncertainty in this range. This pattern is consistent with the well-recognized window of growth faltering that accompanies the transition from exclusive breastfeeding to complementary feeding, when children are at heightened vulnerability to inadequate dietary intake and infectious morbidity.

### 5.6 Maternal BMI

The predicted probability of stunting by maternal BMI is shown in Supplementary Figure S4. Maternal BMI exhibited a nonlinear inverse association with childhood stunting, with the steepest reductions in risk occurring at lower BMI levels. Among children of mothers with BMI below approximately 16 kg/m^2^, the predicted stunting probability was 35.3% (95% CrI: 34.7%−36.0%), indicating substantially elevated risk among children of severely undernourished mothers. As maternal BMI increased, stunting risk declined progressively, reaching 24.9% (95% CrI: 24.2%−25.6%) among children of mothers with BMI between 28 and 35 kg/m^2^, suggesting a protective association of improved maternal nutritional status. At BMI values above approximately 45 kg/m^2^, the predicted probability curve exhibited an apparent upward trend accompanied by substantially wider credible intervals. Given the limited number of observations in this range, this pattern likely reflects data sparsity rather than a meaningful biological relationship and should therefore be interpreted cautiously.

### 5.7 District-level spatial odds ratio of childhood stunting

Supplementary Figure S5 presents the district-level spatial odds ratio (dOR) estimates derived from the spatial interaction model. Marked geographic disparities in adjusted stunting risk were evident across districts. Districts with dOR values greater than 1 exhibited elevated adjusted odds of childhood stunting relative to the national baseline after adjustment for individual-level, maternal, and socioeconomic covariates, net of the overall survey-round effect.

A pronounced concentration of higher-risk districts was observed across parts of central and eastern India, consistent with the strong spatial structure identified by the BYM2 component. In contrast, lower adjusted district-level risks were more frequently observed in southern and western regions. These findings indicate that substantial geographic inequalities in childhood stunting persist even after accounting for observed determinants, highlighting the importance of place-based influences on child nutritional outcomes.

### 5.8 Temporal change between survey rounds

District-level temporal change was estimated directly within the unified Bayesian spatial interaction model, as described in Section 4.4. Supplementary Figure S6 displays the exponentiated district-specific survey-round interaction effects *exp*(δ_*j*_), summarizing district-level temporal change between NFHS-4 (2015–16) and NFHS-5 (2019–21). Although the overall survey-round effect was close to the null value (Table 5), substantial heterogeneity was evident across districts. Some districts demonstrated reductions in adjusted stunting risk, whereas others exhibited increases, indicating that temporal changes were geographically uneven rather than reflecting a uniform national trend.

Using the transition criteria described in Section 4.4, 59 of the 575 harmonized districts demonstrated credible improvement, 35 demonstrated credible worsening, and 481 showed no credible evidence of change between NFHS-4 and NFHS-5 (Table 6; Supplementary Figure S7). The predominance of districts classified as showing no credible change indicates that temporal changes were generally modest relative to estimation uncertainty. Nevertheless, the presence of both improving and worsening districts highlights substantial geographic heterogeneity in temporal trajectories and suggests that progress in reducing childhood stunting has not been uniformly distributed across India. These findings indicate that local contextual factors likely influenced the pace and direction of change across districts.

**Table 6:** District-level temporal change classifications based on district-specific survey-round interaction effects.

Transition Category	Number of districts	Percentage (%)
Credible improvement	59	10.3
No credible change	481	83.7
Credible worsening	35	6.1
Total	575	100.0

Sensitivity analyses using ±5 and ±10% indifference bands around dOR = 1 produced similar overall conclusions, although the proportion of districts classified as indeterminate increased as the threshold widened (Supplementary Tables S2, S3).

### 5.9 Policy prioritization framework

Based on pooled district-level spatial odds ratio (dOR) estimates from the final Bayesian spatial interaction model, districts were first classified into two policy-priority groups according to whether adjusted stunting risk exceeded the national baseline (dOR > 1) or fell below it (dOR < 1). Because the dOR represents a district-specific multiplicative effect on the odds of childhood stunting relative to the national baseline after adjustment for observed covariates and spatial structure, this classification reflects comparative adjusted vulnerability rather than absolute prevalence burden.

Overall, 48.0% of districts (*n* = 276) were classified as higher-priority districts (dOR > 1), indicating above-average adjusted stunting risk, while 52.0% (*n* = 299) were classified as lower-priority districts (dOR < 1) (Table 7). The near-even distribution of districts across the two categories highlights the substantial geographic heterogeneity in childhood stunting risk across India. This pattern was further corroborated by the posterior exceedance probability map (Supplementary Figure S8), which displays P(dOR > 1), the posterior probability that a district's adjusted spatial odds ratio exceeds the national baseline. Districts in central, eastern, and parts of southern India exhibited exceedance probabilities approaching 1.0, reinforcing their classification as higher-priority areas for intervention.

**Table 7:** Policy priority classification based on district-level spatial odds ratio (dOR).

Priority Level	Number of districts	Percentage (%)
High priority (dOR > 1)	276	48.0
Low priority (dOR < 1)	299	52.0
Total	575	100.0

To jointly consider adjusted district-level risk and absolute disease burden, this classification was further refined using a dOR–prevalence quadrant framework. Consistent with standard Bayesian disease-mapping practice, the threshold for elevated adjusted risk was set at dOR = 1, while the prevalence threshold was defined as the national median of district-level NFHS-5 weighted stunting prevalence. Districts were subsequently classified into four categories according to their adjusted risk and observed burden.

A total of 37.0% of districts (*n* = 213) were classified as High dOR & High Prevalence, representing the highest-priority districts for intervention. A further 11.0% (*n* = 63) were categorized as High dOR & Low Prevalence, indicating districts with elevated adjusted vulnerability despite comparatively lower observed burden. An additional 12.9% (*n* = 74) were classified as Low dOR & High Prevalence, suggesting districts where substantial burden persists despite below-average adjusted risk. The remaining 39.1% (*n* = 225) fell within the Low dOR & Low Prevalence quadrant, indicating comparatively lower adjusted risk and burden (Table 8).

**Table 8:** Joint dOR–prevalence quadrant classification of districts.

Quadrant	Number of districts	Percentage (%)
High dOR & high prevalence (top priority)	213	37.0
High dOR & low prevalence	63	11.0
Low dOR & high prevalence	74	12.9
Low dOR & low prevalence	225	39.1
Total	575	100.0

This framework distinguishes districts with both elevated adjusted risk and high observed burden from those where risk and burden are discordant, thereby providing a more nuanced basis for geographic prioritization and resource allocation. Supplementary Figure S9 presents the spatial distribution of the quadrant classifications across India.

## 6 Discussion

This study provides a district-level assessment of childhood stunting in India using a Bayesian spatial geo-additive modeling framework that explicitly accounts for both spatial heterogeneity and district-specific temporal change. Although India has experienced substantial investments in maternal and child nutrition over the past decade, the findings indicate that progress has not been geographically uniform. After accounting for district-specific temporal variation, changes in stunting risk differed considerably across districts rather than following a consistent national trajectory. This finding highlights the limitations of relying solely on national averages to assess progress and underscores the importance of district-level analyses for identifying persistent geographic inequalities.

The substantial improvement in model performance achieved through the inclusion of spatial random effects confirms that childhood stunting remains strongly patterned by geography. Geographically proximate districts tended to share similar adjusted risk profiles even after accounting for observed socioeconomic characteristics, suggesting that contextual factors such as food environments, sanitation infrastructure, health-service accessibility, social conditions, and broader development patterns contribute to geographic inequalities in child nutrition. These findings are consistent with previous research demonstrating that place-based determinants influence childhood undernutrition beyond individual and household-level factors.

Further examination of the spatial structure indicated that the geographic clustering of stunting risk was driven primarily by spatially correlated processes shared across neighboring districts rather than by isolated district-specific factors, consistent with decomposition of the pooled BYM2 spatial random effect, which demonstrated substantially greater variation in the structured spatial component than in the unstructured component (Supplementary Figures S10–S11). This pattern suggests that broader regional and contextual influences continue to shape childhood nutritional outcomes across large areas of the country. Consequently, interventions focused exclusively on individual or household characteristics may be insufficient unless accompanied by improvements in the wider social, environmental, and service-delivery context.

The nonlinear association between child age and stunting risk identified a critical vulnerability window during the first two years of life. Risk increased during the transition from exclusive breastfeeding to complementary feeding, a period often characterized by inadequate dietary diversity, suboptimal feeding practices, and increased exposure to infectious diseases. The persistence of this pattern in a nationally representative dataset reinforces the importance of strengthening nutrition interventions during the first 1,000 days of life.

Maternal nutritional status also emerged as an important determinant of childhood stunting. The nonlinear inverse relationship between maternal BMI and stunting risk indicates that maternal undernutrition substantially increases the likelihood of impaired child growth. These findings support the growing body of evidence emphasizing the intergenerational transmission of nutritional disadvantage and underscore the importance of interventions that improve maternal nutrition before conception, during pregnancy, and throughout early childhood. These findings further suggest that important biological relationships may be overlooked when key predictors are modeled using simple linear assumptions.

Direct estimation of the district-specific survey-round interaction effect revealed substantial geographic heterogeneity in temporal trajectories of childhood stunting. The observed variation in district-level temporal trajectories indicates that progress has not been evenly distributed across the country. These findings suggest that local contextual factors continue to influence the effectiveness of nutrition and health interventions and reinforce the value of district-specific monitoring for evaluating policy impact.

Although temporal changes varied across districts, broad geographic patterns of elevated risk remained relatively stable over time. This persistence suggests that district-level stunting risk continues to be influenced by long-standing structural determinants, including poverty, maternal undernutrition, health-service access, sanitation infrastructure, and food security conditions documented in previous studies.

To support policy planning, adjusted district-level risk was considered jointly with observed district-level stunting prevalence. This integrated perspective helps distinguish districts facing both elevated vulnerability and substantial burden from those where risk and burden are less closely aligned. Such an approach provides a practical basis for geographic targeting and resource allocation, allowing interventions to be directed toward areas where both underlying vulnerability and population burden remain greatest.

## 7 Limitations

Several limitations should be considered when interpreting these findings. Although NFHS sampling weights were incorporated into all models, the full multistage survey design was not propagated through variance estimation; consequently, posterior credible intervals for fixed-effect estimates may be somewhat narrower than those obtained under a fully design-based approach.

The covariate set was restricted to variables available with adequate completeness across both survey rounds. Important determinants of childhood stunting—including sanitation, water access, dietary diversity, breastfeeding practices, birth order, maternal anemia, caste or social group, and rural–urban residence—were not included. Although some of their geographic variation may be partially captured by the district-level spatial effects, residual confounding cannot be excluded.

The temporal analysis was constrained by the availability of only two cross-sectional survey rounds. While district-specific survey-round interaction effects allowed temporal change to be estimated directly within a unified modeling framework, the limited temporal replication precluded estimation of more complex spatio-temporal dependence structures and long-term district trajectories. Consequently, temporal patterns should be interpreted as changes between two survey rounds rather than continuous temporal processes.

District harmonization restricted the analysis to 575 consistently defined districts, excluding areas created through administrative subdivision between 2015 and 2021. These excluded districts may differ systematically from those retained in the analysis. In addition, the spatial adjacency graph contained isolated districts and disconnected sub-graphs, which may have modestly influenced spatial variance estimates despite the ability of the BYM2 model to accommodate such structures.

Finally, multiple children from the same household could contribute to the analysis. Because household-level random effects were not explicitly modeled, some within-household correlation may remain, potentially resulting in slightly narrower credible intervals. However, point estimates are unlikely to be materially affected.

## 8 Conclusions

Childhood stunting remains a major public health challenge in India, with substantial geographic inequalities persisting across districts. Using a Bayesian spatial geo-additive modeling framework incorporating district-specific temporal interactions, this study demonstrates that childhood stunting is strongly patterned by geography and that temporal changes between NFHS-4 and NFHS-5 have been highly heterogeneous across districts.

The findings indicate that residual variation in childhood stunting remains strongly geographically structured, suggesting that contextual influences operating beyond individual and household characteristics continue to shape nutritional outcomes across districts. Persistent geographic inequalities and uneven temporal progress highlight the importance of incorporating spatial context into nutrition surveillance and intervention planning.

The findings underscore the need for district-specific strategies that prioritize maternal nutrition and interventions during the first 1,000 days of life. By integrating district-level adjusted risk and observed stunting prevalence, the dOR–prevalence framework provides a practical basis for identifying areas requiring intensified intervention and resource allocation. Spatially targeted policies informed by district-level risk profiles are likely to be essential for achieving sustained and equitable reductions in childhood stunting across India and for accelerating progress toward national nutrition goals.

## 9 Future work

Future work should extend the present framework through the incorporation of additional NFHS rounds or other longitudinal data sources, enabling the development of fully spatio-temporal Bayesian models capable of estimating district-level temporal risk trajectories across multiple survey rounds. Incorporating the unmeasured determinants noted in the Limitations section and additional contextual information would further improve understanding of the mechanisms underlying geographic disparities in childhood stunting and help reduce residual spatial confounding.

Further methodological extensions could incorporate household-level random effects to account explicitly for within-household clustering and provide more accurate uncertainty quantification. Additional model validation through comprehensive posterior predictive checks and residual diagnostics would also strengthen inference and complement the strong agreement observed between model-predicted and survey-estimated district prevalence.

## Data Availability

The original contributions presented in the study are included in the article/Supplementary material, further inquiries can be directed to the corresponding author.
