# Supplementary material for: Addressing the poverty–nutrition gap for SDG 1 and SDG 2: a Bayesian geo-additive analysis of spatial heterogeneity and nonlinear maternal risks in childhood stunting in India (NFHS-4 and NFHS-5)
Source: Front Public Health. 2026 Aug 3;14:1854301. doi: 10.3389/fpubh.2026.1854301 (PMC13478098; doi:10.3389/fpubh.2026.1854301)
Supplement: Supplementary file 1 [file Supplementary_File_1.docx]

Supplementary Material

# 1 **Supplementary Figures and Tables**

## 2 **Supplementary Figures**

**Supplementary Graphical Abstract.** Overview of the Bayesian spatial geo-additive modelling framework used to investigate district-level spatial heterogeneity, nonlinear effects of child age and maternal body mass index (BMI), and district-specific temporal variation in childhood stunting across 575 harmonized districts in India using NFHS-4 (2015–16) and NFHS-5 (2019–21). The framework integrates BYM2 spatial random effects, second-order random walk (RW2) smoothers, and district-specific survey-round interaction effects within a unified Bayesian model to identify geographic inequalities, quantify temporal heterogeneity, and prioritize districts for targeted intervention.

**Supplementary Figure S1.** Model calibration plot comparing observed district-level stunting prevalence (weighted survey estimates) with model-predicted stunting probabilities across the 575 harmonized districts.

**Supplementary Figure S2**. Model-based comparison of mean predicted probability of childhood stunting between NFHS-4 (2015–16) and NFHS-5 (2019–21). Error bars represent 95% posterior credible intervals

**Supplementary Figure S3.** Predicted probability of childhood stunting across child age (months). The solid line represents the posterior mean predicted probability of childhood stunting evaluated at the population-average covariate profile, and the shaded bands indicate 95% posterior credible intervals.

**Supplementary Figure S4.** Predicted probability of childhood stunting across maternal body mass index (BMI). The solid line represents the posterior mean predicted probability of childhood stunting evaluated at the population-average covariate profile, and the shaded bands indicate 95% posterior credible intervals.

**Supplementary Figure S5.** District-level spatial odds ratio $(dOR = exp(b_{j}))$ of childhood stunting estimated from the final Bayesian spatial interaction model. Values greater than 1 indicate elevated adjusted stunting risk relative to the national baseline (reference levels of all covariates, pooled across survey rounds). This quantity is round-invariant and does not include district-specific temporal effects.

**Supplementary Figure S6.** District-specific temporal interaction in adjusted childhood stunting risk between NFHS-4 (2015–16) and NFHS-5 (2019–21), expressed as $exp(\delta_{j})$, the exponentiated district-specific survey-round interaction effect from the Bayesian spatial interaction model. Values greater than 1 indicate a relative increase, whereas values less than 1 indicate a relative decrease in adjusted childhood stunting odds from NFHS-4 to NFHS-5, after accounting for the overall national temporal trend.

**Supplementary Figure S7.** Classification of district-level temporal change based on district-specific survey-round interaction effects. Districts are classified as showing credible improvement, credible worsening, or no credible change according to 95% Bayesian credible intervals.

**Supplementary Figure S8.** Posterior exceedance probability of elevated childhood stunting odds across Indian districts from the final Bayesian spatial interaction model. The map displays P(dOR > 1), the posterior probability that the pooled district-level spatial odds ratio exceeds the national baseline (reference levels of all covariates, pooled across survey rounds).

**Supplementary Figure S9.** Geographic distribution of districts across the four dOR–prevalence quadrants. Districts classified as “High dOR & High Prevalence” represent the highest-priority areas for intervention.

**Supplementary Figure S10.** Spatially structured (ICAR) component of the district-level BYM2 random effect, decomposed from the final Bayesian spatial interaction model.

Note: Represents residual spatial heterogeneity only; not a temporal quantity.

**Supplementary Figure S11.** Unstructured component of the district-level BYM2 random effect, decomposed from the final Bayesian spatial interaction model.

Note: Represents residual spatial heterogeneity only; not a temporal quantity.

## 3 **Supplementary Tables**

**Supplementary Table S1.** Variance inflation factors (VIFs) for fixed-effect covariates included in the Bayesian spatial interaction model.

| **Variable** | **VIF** |
| --- | --- |
| Household wealth quintile | 1.331 |
| Maternal education | 1.351 |
| Child Sex | 1.000 |
| Survey round | 1.022 |

**Supplementary Table S2.** Sensitivity analysis of threshold-based district classifications using a ±5% indifference band around dOR = 1.

| **Transition category** | **Districts (n)** | **Percentage (%)** |
| --- | --- | --- |
| Improved | 35 | 6.1 |
| Indeterminate — NFHS-4 | 68 | 11.8 |
| Indeterminate — NFHS-5 | 80 | 13.9 |
| Indeterminate — both rounds | 19 | 3.3 |
| Stagnant | 334 | 58.1 |
| Worsened | 39 | 6.8 |
| Total | 575 | 100.0 |

**Supplementary Table S3.** Sensitivity analysis of threshold-based district classifications using a ±10% indifference band around dOR = 1.

| **Transition category** | **Districts (n)** | **Percentage (%)** |
| --- | --- | --- |
| Improved | 11 | 1.9 |
| Indeterminate — NFHS-4 | 89 | 15.5 |
| Indeterminate — NFHS-5 | 113 | 19.7 |
| Indeterminate — both rounds | 86 | 15.0 |
| Stagnant | 259 | 45.0 |
| Worsened | 17 | 3.0 |
| Total | 575 | 100.0 |
